# Mitochondrial haplogroup N1a phylogeography, with implication to the origin of European farmers

**DOI:** 10.1186/1471-2148-10-304

**Published:** 2010-10-12

**Authors:** Malliya Gounder Palanichamy, Cai-Ling Zhang, Bikash Mitra, Boris Malyarchuk, Miroslava Derenko, Tapas Kumar Chaudhuri, Ya-Ping Zhang

**Affiliations:** 1 Laboratory for Conservation and Utilization of Bio-resources, Yunnan University, 2 North Green Lake Street, Kunming 650091, China; 2 State Key Laboratory of Genetic resources and Evolution, Kunming Institute of Zoology, Chinese Academy of Sciences, Kunming 650223, China; 3 Cellular Immunology Laboratory, Department of Zoology, University of North Bengal, Siliguri 734013, West Bengal, India; 4 Institute of Biological Problems of the North, Far-East Branch of the Russian Academy of Sciences, Magadan 685000, Russia

## Abstract

**Background:**

Tracing the genetic origin of central European farmer N1a lineages can provide a unique opportunity to assess the patterns of the farming technology spread into central Europe in the human prehistory. Here, we have chosen twelve N1a samples from modern populations which are most similar with the farmer N1a types and performed the complete mitochondrial DNA genome sequencing analysis. To assess the genetic and phylogeographic relationship, we performed a detailed survey of modern published N1a types from Eurasian and African populations.

**Results:**

The geographic origin and expansion of farmer lineages related N1a subclades have been deduced from combined analysis of 19 complete sequences with 166 N1a haplotypes. The phylogeographic analysis revealed that the central European farmer lineages have originated from different sources: from eastern Europe, local central Europe, and from the Near East via southern Europe.

**Conclusions:**

The results obtained emphasize that the arrival of central European farmer lineages did not occur via a single demic diffusion event from the Near East at the onset of the Neolithic spread of agriculture into Europe. Indeed these results indicate that the Neolithic transition process was more complex in central Europe and possibly the farmer N1a lineages were a result of a 'leapfrog' colonization process.

## Background

The emergence of farming in the Near East and its spread to Europe is one of the most significant processes in the evolutionary history of Europeans. The question of how agriculture spread into Europe and associated with the genesis of the genomes of modern Europeans has been a subject of debate and controversy among archaeologists, anthropologists, and geneticists. Some have argued that agriculture was introduced in large parts of Europe by rapid movement of immigrant farmers from the Near East [1]. Others have postulated the indigenous development of farming by hunter-gatherers and only minimal immigration from outside [2]. Recently researchers have taken steps to address this issue through direct comparison of ancient mitochondrial DNA (mtDNA) from European hunter-gatherers and early farmers [3,4].

Farming practices spread across Europe after the domestication of plants and animals in the Near East around 12,000 years ago. Archaeologists note that the spread of farming into central Europe was accompanied by Linear Pottery (Linearbandkeramik - LBK) and Alfödi Linear Pottery Culture (AVK). These early farming cultures are thought to have originated in Hungary and Slovakia about 7,500 years ago from where they spread rapidly as far as the Paris Basin and the Ukraine. To assess the Neolithic female contribution and associated farming spread into central Europe we performed a genetic study on the farmers skeletons remains from the LBK/AVK area. The first hypervariable segment (HV1) of the mtDNA control sequences information obtained from 24 out of 57 Neolithic skeletons from various LBK/AVK locations in Germany, Austria and Hungary belonged to typical western Eurasian haplogroups (H or V, T, K, J, U3, and N1a). Among them, six of the farmers' skeletons had a distinctive and rare N1a mtDNA type. Furthermore, five of these six individuals display different N1a haplotypes and they were widespread in the LBK area. But today this N1a mtDNA type is very rare (0.2%) in Europeans, suggesting that first Neolithic farmers did not have a strong impact on the genetic population structure of the modern European female lineages. Thus, this study result suggests that small pioneer farming groups carried farming into new areas of Europe and the surrounding hunter-gatherers adopted the new culture and then outnumbered the original farmers, diluting their N1a frequency to the low modern value [3].

However, previous studies did not address the question whether the mtDNA N1a-lineages of the skeletons belonged to immigrant farmers or originated from local Mesolithic peoples who adopted the farming [5]. Recently, researchers have presented important new data on this question by direct comparison of ancient mtDNAs from European hunter-gatherers and early farmer skeletons [4]. The most striking result of this study is that the mtDNA sequences of the early farmers were genetically distinct from the hunter-gatherers, thus confirming the hypothesis that the first farmers were not the descendants of local hunter-gatherers but immigrated into central Europe. Thus, it is concluded that outside colonizers brought farming to central Europe by a major migration event [4,6]. Furthermore, the craniometric data of the Mesolithic and Neolithic skull from Southwest Asia and Europe also suggest that the spread of farming into Europe was mainly due to the active dispersal of people from Southwest Asia [7].

Nevertheless, Rowley-Conwy [5] postulated the appearance of farming in Europe was not a demic "wave of advance" from the Near East, but rather a combination of small-step migrations, in which indigenously agriculturalised hunter-gatherers played an important role in the spread of farming in Europe. Further, he emphasized that the non-local mtDNA lineages of central Europe's earliest farmers most likely were of Greek, Balkan, or Black Sea hunter-gatherer descent rather than of the Near East origin. In addition, both central European farmer haplotypes and the N1a haplotypes found in the modern Europeans were not found in the Near East region, thus suggesting that the farmer N1a lineages may be indigenous to central Europe and less likely that they were originated from the Near East agriculturists [8]. To clarify the inconsistency on the farmers' origin and associated farming spread into central Europe, more detailed examination of farmers N1a lineages is required.

Our combined mtDNA-HV1 network analysis of farmer and modern N1a types reveals three phylogeographic branches- European, Central Asian, and African/South Asian. The LKB farmer skeleton individuals- Derenburg 1, 3 (DEB1 & 3), Halberstadt 2 (HAL2), Flowborn 1 (FLO1), and Unterwiederstedt 5 (UWS5) types falls into the European N1a sub-branch. The AVK individual- Ecsegfalva 1 (ECS1) belongs to the Central Asian sub-branch. The African/South Asian branch is characterized by control region motif 16147G, which differs from the European and the Central Asian branches with the 16147A variant. However, a phylogeographic pattern based only on the HV1 sequence variation is insufficient to reveal the detailed genetic history of European farmers. On the contrary, the advance of complete mtDNA sequence analysis seems to be extremely useful for maternal phylogeny of farmer individuals reconstruction and thus may shed light on their origins and genetic history; furthermore, it helps to understand the movements of Neolithic communities across Europe in the past.

In order to reconstruct an unambiguous phylogeny of the farmer N1a types, 12 mtDNAs (from 2 Russia, 1 Tatar- Russia, 1 Kazakh- South Siberia, 1 Hungary, 1 France, 1 Italy, 2 India, and 3 European ancestry living in United States of America) were completely sequenced and compared with 7 previously published complete mtDNA N1a sequences. Our sampling strategy was based on the preliminary obtained data on HV1 sequence variation; in particularly samples closely related to the farmers' N1a haplotypes were chosen for complete mtDNA sequencing from the variety of modern populations (further details refer the materials and methods). Further, based on farmer lineage subclades information obtained, we have attempted to trace the possible areas of their origin by comparison with all available so far data from the European, Central Asian, Near Eastern, and South Asian populations as well as from the Caucasus.

## Results and Discussion

**Figure 1:**
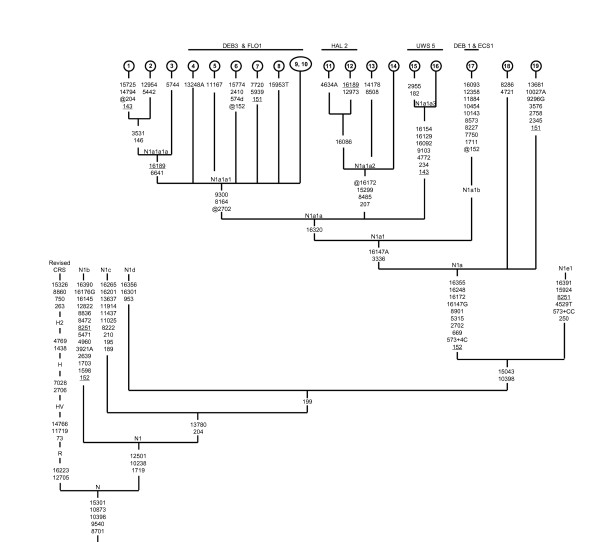
**Haplogroup N1a phylogeny**. The tree is rooted in macrohaplogroup N. Mutations are scored relative to the revised Cambridge reference sequence (rCRS) [30]. The prefix "@" indicates back mutation, recurrent mutations are underlined, transversions have a base suffix, "d" deletions and "+" insertions, and the poly(C) region in HVS1 and -2 as well as 16519 is excluded. Sequences 1-2, 7-12, 14-17, are new while others have been previously reported [9,31-33]. Samples names are denoted by numerals and their origin is described in the material and methods section. The central European farmer samples N1a type affinities with respective subclades are mentioned on the top of the tree.

A tree of the 19 complete mtDNA N1a sequences is presented in Figure 1, which includes also 7 previously published sequences. The phylogeny reveals an early split of African/Near East lineages with the HV1 region variant 16147G from the European and Central Asian lineages, which carry the 16147A variant. In addition, later lineages share a coding region transition at nucleotide position (np) 3336, which allows the definition of subhaplogroup N1a1. Within the subhaplogroup N1a1, we noticed that the farmer samples - DEB1, ECS1 and other published sequences from Arabian Peninsula, Armenia, Egypt, and Setoland lacks the HV-1 mutation 16320. We sequenced a single mtDNA genome (sample 17, figure 1) belonging to this group and the results have allowed us to rename N1a1 of Derenko et al. [9] as N1a1a. Now the revised phylogenetic tree points to 3336 and 16147A as the basal mutations for N1a1 subhaplogroup, whereas transition 16320 seems to be a characteristic mutation for a whole N1a1a. We have named the new haplotype sequence as N1a1b. Furthermore, previously established subgroup N1a1a defined by mutations 8164, 9300, and a back mutation 2702 were absent in the lineages 11 to 16. This suggests that 2702, 8164 and 9300 are diagnostic mutations for one of the N1a1a subbranches which is named N1a1a1 here [10]. Finally, our new data identify two additional subclades that have not been reported earlier, these lineages are designated as N1a1a2 and N1a1a3 (Figure 1).

From our complete mtDNA sequences phylogeny, five different mtDNA haplotypes were observed within the central European farmers by Haak et al. [3] were classified into four subhaplogroups (Figure 1). The farmer samples DEB3 and FLO1 have an identical HV-1 motif 16147A-16172-16223-16248-16320-16355. The results of phylogenetic analysis of complete mtDNA sequences exactly matched to modern samples suggest that the farmer DEB3 and FLO1 lineages might belong to subhaplogroup N1a1a1. The exactly matched sequence with farmer HAL2 N1a haplotype (16086-16147A-16172-16223-16248-16320-16355) was observed in modern Portuguese population from south Portugal. Nevertheless, in combination with specific HV-2 motif 152-199-204-207, a subset of closely related sequences was found in the modern populations and results of complete mtDNA sequencing of some of those samples (samples 11 and 12, Figure 1) unambiguously placed the farmer HAL2 lineage into subhaplogroup N1a1a2. Comparing the HV-1 motif of farmer sample UWS5 with the modern complete mtDNA sequence phylogeny (sample 15 and 16, figure 1) suggests that the farmer UWS5 lineage belong to subhaplogroup N1a1a3. Finally, farmer lineages DEB1 and ECS1 lacking the HV1 mutation 16320 belong to N1a1b branch.

**Figure 2:**
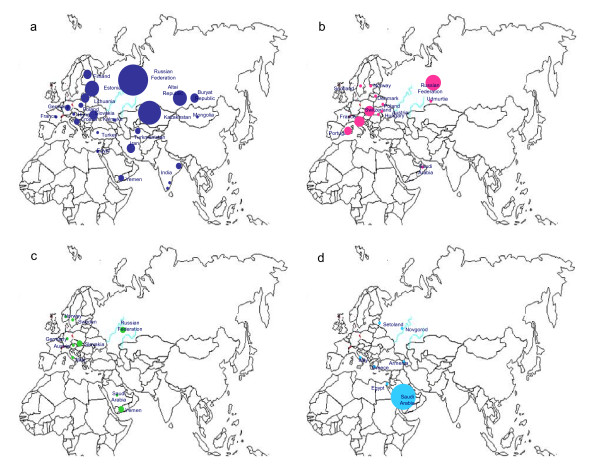
**Modern geographical spread of the farmers related N1a1 subclades:**. a & b depict the eastern and central European origin of the farmer DEB3, FLO1 and HAL2 types affiliated N1a1a1 and N1a1a2 lineages distribution; c & d representing the farmer UWS5 and DEB1 & ECS1 related N1a1a3 and N1a1b subclades haplotypes distribution in the Near East and southern Europe region. Circle areas are proportional to haplotype frequencies.

In order to obtain a detailed picture of the central European farmers N1a lineage origin, we have performed a systematic phylogeographic survey by comparison of mtDNA sequences associated with farmer lineages with the published modern Eurasian data set and the results of this survey are reported in Table 1. As seen, mtDNA sequences of subcluster N1a1a1 related to the farmer DEB3 and FLO1 lineages are widely distributed, accounting for nearly half (82 out of 166) of the total N1a data (Table 1). Lineages from this subcluster were observed in populations of eastern/central Europe, Volga-Ural region, central Asia, South Siberia, Egypt, Yemen, Iran, Turkey, and eastern/southern India (Figure 2a) [3,9,11-16]. The cumulative frequency of N1a1a1 reaches a maximum in eastern Europe and central Asia, and decreases in the direction of central and northern Europe. The same occurs in the southern direction, towards Anatolia and the Caucasus. Furthermore, one of the N1a1a1 subclade, N1a1a1a, characterized by 16189 and 6641 in the HV1 and coding region is restricted to Kazakhstan, Altai and Buryat Republic, and European part of Russian Federation [3,9,14,16-19], and it shows significant heterogeneity (n = 42; χ^2 ^= 125.995; d. f. 81; p < 0.001) in the haplotype distribution, attesting that these areas were a center of expansion. The calculated expansion times of N1a1a1 and N1a1a1a were between 6,800 - 10,600 years (Table 2). Sequences from another N1a1a2 subcluster related to farmer HAL2 haplotype were observed in Denmark, Poland, Scotland, Norway, Switzerland, France, Portugal, Hungary, Austria, and Volga-Ural region [3,19-22] (Figure 2b). This subcluster shows a very young coalescence age between 3,400 - 4,000 years (Table 2). Overall, the observed phylogeographic distribution of farmer lineage related subclades N1a1a1 and N1a1a2 suggests that the ancient farmer sample DEB3, FLO1 and HAL2 may have been associated with local central and eastern European origin rather than with diffusion from Near East.

**Table 1:** Distribution of mtDNA-N1a haplotypes

Haplogroup	HVS-I(minus 16000)^a^	HVS-II (73, 263 in addition)^b^	N	Sample origin	**References**[3]^c^
N1a	147G-172-223-248		1	Turkey	[3]
N1a	147G-172-223-248-355		1	Yemen	[24]
N1a	147G-172-223-248-355		5	Ethiopia	[3]
N1a	147G-172-223-248-355		2	Tanzania	[3]
N1a	147G-172-223-248-355	151-199-204	2	Arabia Peninsula	[20]
N1a	147G-172-223-248-355	151-199-204	1	Saudi Arabia	[23]
N1a	147G-172-223-248-355	199-204	1	Arabia Peninsula	[20]
N1a	147G-172-223-248-355	199-204	1	Dubai-Arabia	[26]
N1a	147G-172-223-248-355	194-199-204	1	Bedouin-Israel	[25]
N1a	147G-172-223-248-355	152-199-204	1	Greece	[27]
N1a	147G-172-223-248-355	152-199-204	1	Russia	[19]
N1a	147G-172-223-224-248-355-357		1	Greece	[3]
N1a	147G-172-223-248-260-355		1	Ethiopia	[3]
N1a	147G-223-248-263-266-355		2	Yemen	[3]
N1a	147G-172-223-248-263-266-355		1	Yemen	[3]
N1a	147G-172-213-223-248-355		1	Somali	[3]
N1a	147G-172-213-223-248-291-355	146-152-182-185C	1	Ethiopia	[25]
N1a	124-147G-172-213-223-248-355		1	Yemen	[3]
N1a	147G-172-223-248-295-355		3	India - South West	[3]
N1a	147G-172-209-223-248-295-355		2	India - South West	[3]
N1a	147G-172-223-248-295-297-355		1	Kabardian, North Caucasus	[3]
N1a	147G-172-223-248-295-297-344-355		1	Turkey	[3]
N1a	147G-172-223-248-295-344-355		1	Iran	[13]
N1a1a1	172-223-248-320-355		1	Lithunia	[14]
N1a1a1	147A-172-248-320-355		1	Poland	[3]
N1a1a1	147A-172-223-248-320		1	Estonia	[3]
N1a1a1	147A-172-223-248-320		1	Tatar	[3]
N1a1a1	147A-172-223-248-320-355		2	Yemen	[3]
N1a1a1	147A-172-223-248-320-355		1	Turkmenistan	[3]
N1a1a1	147A-172-223-248-320-355		1	Iran	[13]
N1a1a1	147A-172-223-248-320-355		1	Kazakhstan	[16]
N1a1a1	147A-172-223-248-320-355		2	Russia	[3]
N1a1a1	147A-172-223-248-320-355		1	Estonia	[14]
N1a1a1	147A-172-223-248-320-355		5	Estonia	[3]
N1a1a1	147A-172-223-248-320-355		1	Lithunia	[14]
N1a1a1	147A-172-223-248-320-355		1	Iran	[3]
N1a1a1	147A-172-223-248-320-355		1	Slovakia	[3]
**N1a1a1**	147A-172-223-248-320-355	152-199-204	1	Tatar-Russia	Present study (9)
**N1a1a1**	147A-172-223-248-320-355	152-199-204	1	Russia	Present Study (10)
N1a1a1	147A-172-223-248-320-355	152-199-204	1	Slovakia	[15]
N1a1a1	147A-172-223-248-320-355	152-199-204	1	Altai Republic	[9]
N1a1a1	147A-172-223-248-320-355	152-199-204	3	Finland	[14]
**N1a1a1**	147A-172-223-248-320-355	152-199-204	2	India	Present study (8)
**N1a1a1**	147A-172-223-248-320-355	151-152-199-204	1	India	Present study (7)
N1a1a1	147A-172-223-248-291-320-355		1	Egypt	[3]
N1a1a1	147A-172-223-248-294-320-355		1	Estonia	[3]
N1a1a1	147A-172-223-248-294-320-355		1	India - South East	[3]
N1a1a1	147A-172-223-248-295-320-355		1	Buryat Republic	[3]
N1a1a1	147A-172-209-223-248-320-355		2	Croation-Italian	[3]
N1a1a1	147A-172-206-223-248-320-355		1	France	[3]
N1a1a1	147A-172-195-223-248-320-355		1	Germany	[3]
N1a1a1	114A-147A-172-223-248-320-325-355		1	Germany	[3]
N1a1a1	93-147A-169-172-193iC-223-248-320-355		1	Russia	[12]
N1a1a1a	147A-189-223-248-272-320-355		1	Russia	[3]
N1a1a1a	147A-172-189-223-248-320-355		1	Russia	[17]
N1a1a1a	147A-172-189-223-248-320-355		1	Russia	[18]
N1a1a1a	147A-172-189-223-248-320-355		5	Kazakhstan	[16]
N1a1a1a	147A-172-189-223-248-320-355		1	Hungary	[3]
N1a1a1a	147A-172-189-223-248-320-355		6	Russia	[3]
N1a1a1a	147A-172-189-223-248-320-355		3	Altai Republic	[3]
N1a1a1a	147A-172-189-223-248-320-355		1	Kalmyks	[3]
N1a1a1a	147A-172-189-223-248-320-355		5	Kazakhs	[3]
N1a1a1a	147A-172-189-223-248-320-355		3	Turkey	[3]
N1a1a1a	147A-172-189-223-248-320-355		1	Hungary	[3]
N1a1a1a	147G-172-189-223-248-320-355		1	Altai Republic	[3]
N1a1a1a	147A-172-189-223-248-270-320-355		1	Lithunia	[14]
N1a1a1a	147A-172-189-223-248-272-320-355		4	Russia	[3]
N1a1a1a	147A-172-183C-189-223-248-320-355		1	Buryat Republic	[3]
N1a1a1a	147A-172-189-193iC-223-248-320-355	152-199-204	1	Poland	[19]
N1a1a1a	147A-172-189-193iC-223-248-320-355	152-199-204	1	Ulaanbaatar	[9]
N1a1a1a	147A-172-189-193iC-223-248-320-355	152-199-204	1	Buryat Republic	[9]
**N1a1a1a**	147A-172-189-193iC-223-248-320-355	143-146-152-199	1	Kazakh-South Siberia	Present study (1)
**N1a1a1a**	147A-172-189-193iCC-223-248-320-355	146-152-199-204	1	Hungary	Present study (2)
N1a1a1a	147A-172-187A-189-223-248-320-355		1	Russia	[3]
N1a1a1a	147A-172-181-189-223-248-320-355		1	Kazakhstan	[16]
N1a1a2	147A-223-248-320-355		1	France	[3]
**N1a1a2**	147A-223-248-320-355	152-199-204-207	1	United States of America	Present Study (14)
N1a1a2	147A-172-223-248-320-355	152-199-204-207	1	Poland	[19]
N1a1a2	86-147A-223-320-355		1	Switzerland	[3]
**N1a1a2**	86-147A-223-248-320-355	152-199-204-207	1	Arizona	Present Study (11)
N1a1a2	86-147A-223-248-320-355		3	Switzerland	[3]
N1a1a2	86-147A-223-248-320-355	152-199-204-207	1	Austria	[21]
N1a1a2	86-147A-189-223-248-320-355		1	Denmark	[3]
**N1a1a2**	86-147A-189-193iCC-223-248-320-355	152-199-204-207	1	France	Present Study (12)
N1a1a2	86-147A-223-248-319-320-355		1	Scotland	[3]
N1a1a2	86-147A-223-248-320-324-355		1	Acores-Portugal	[3]
N1a1a2	86-147A-223-248-278-320-355		6	Russia	[3]
N1a1a2	86-147A-223-248-278-320-355		1	Udmurts	[3]
N1a1a2	86-147A-148-214-223-320-355		1	Norway	[3]
N1a1a2	86-147A-172-223-248-320-355		1	Portugal	[22]
N1a1a2	86-147A-172-223-248-320-325-355	152-199-204-207	1	Arabia Peninsula	[20]
N1a1a2	86-147A-164-172-223-248-320-355		1	France	[3]
N1a1a2	86-147A-164-172-223-248-320-355		1	Portugal	[3]
N1a1a2	86-147A-164-172-223-248-320-355		1	Hungary	[3]
N1a1a2	86-147A-150-164-172-209-223-248-320-355-463	152-199-204-207	1	New Mexico	Present Study
N1a1a3	147A-154-172-223-320-355	152-199-204	1	Austria	[21]
N1a1a3	147A-154-172-223-320-355		1	Sweden	[3]
N1a1a3	147A-154-172-223-320-355		1	Slovakia	[3]
N1a1a3	147A-154-172-223-320-355	152-199-203-204	1	Slovakia	[15]
N1a1a3	147A-154-172-223-248-258-320-355	152-199-204	1	Arabia Peninsula	[20]
N1a1a3	147A-154-170-172-223-248-320-355		2	Yemen	[3]
**N1a1a3**	92-129-147A-154-172-223-248-320-355	143-152-182-199-204-234	1	South Carolina	Present Study (15)
**N1a1a3**	92-129-147A-154-172-223-248-320-355	143-152-199-204-234	1	Russia	Present Study (16)
N1a1a3	92-129-147A-154-172-223-248-320-355		1	Molise-Italy	[3]
N1a1a3	92-129-147A-154-172-223-248-320-355		1	Norway	[3]
N1a1a3	92-129-147A-154-172-223-248-320-355		1	Germany	[3]
N1a1b	147A-172-223-248-355		1	Setoland	[14]
N1a1b	147A-172-223-248-355		1	Egypt	[3]
N1a1b	147A-172-223-248-355		1	Armenia	[3]
**N1a1b**	93-147A-172-223-248-355	199-204	1	Italy	Present Study (17)
N1a1b	147A-172-223-245-248-355	183-199-204	1	Arabia Peninsula	[20]
N1a1b	147A-172-189-223-248-355	199-204	1	Arabia Peninsula	[20]
N1a1b	147A-172-223-248-266-274-355	199-204	1	Arabia Peninsula	[20]
N1a1b	147A-172-218-223-248-261-274-355	41-199-204	1	Arabia Peninsula	[20]
N1a1b	147A-172-218-223-248-261-274-355	41-199-204	4	Saudi Arabia	[23]

Mutations are scored relative to the rCRS [30]. Transversions and length variants (i = insertion) are specified.

^a^The sequences shown in bold letter are studied by us. Number shown in parentheses corresponds to sequence number in the phylogenetic tree (Figure 1)

^b^Length variation in the 303-309 and 311-315 C-stretch region is ignored.

^c^References therein Haak et al. [3].

**Table 2:** Estimated ages (years) for different sub-clades of N1a haplogroup

Clade	No. of mtDNAs	all coding-region base substitutions		only synonymous transitions			
			
		age^a^	age^b^	age^c^	age^b^	age^d^	age^e^
N1a	19	23,541 ± 6,476	21,114 ± 5,809	19,616 ± 7,576	22,185 ± 8,568	22,864 ± 8,830	23,171 ± 8,949
N1a1	17	18,761 ± 5,037	16,827 ± 4,518	13,934 ± 5,005	15,759 ± 5,661	16,241 ± 5,834	16,459 ± 5,913
N1a1a1	10	8,738 ± 2,570	7,837 ± 2,305	6,764 ± 2,841	7,650 ± 3,213	7,884 ± 3,311	7,990 ± 3,356
N1a1a1a	3	11,976 ± 5,140	10,741 ± 4,610	8,996 ± 5,546	10,175 ± 6,273	10,486 ± 6,465	10,627 ± 6,552
N1a1a2	4	5,140 ± 2,570	4,610 ± 2,305	3,382 ± 2,367	3,825 ± 2,678	3,942 ± 2,759	3,995 ± 2,797

a. - mutation rate of Mishmar et al. [35]

b. - mutation rate of Perego et al. [37]

c. - mutation rate of Kivisild et al. [36]

d. - mutation rate of Soares et al. [38]

e. - mutation rate of Loogvali et al. [39]

The farmer UWS5 lineage associated with subclade N1a1a3 which is widespread in Italy, Yemen, Arabian Peninsula, Austria, Germany, Slovakia, Sweden, and Norway (Figure 2c) [15,20,21]. So, based on the current distribution of subcluster N1a1a3 haplotypes, it is rather difficult to establish the exact geographic origin of the farmer sample UWS5. Nevertheless, the preferential distribution of this haplotype in the Near East and central/western Europe as well as its scarce observation in eastern Europe suggests that the lineage ancestral to UWS5 dispersed probably from the Near East through the southern Europe and then into central/western Europe. However, the clarification of the most likely place of origin of farmer UWS5 lineage requires further study. The farmer lineages ECS1 and DEB1 belong to sub-branch N1a1b which is found in the Arabian Peninsula, Armenia, and Italy [3,14,20,23] (Figure 2d). Thus, the presence of this lineage in central Europe may represent a Near Eastern influence due to both a high frequency and a high degree of diversity of this lineage in the Arabian Peninsula.

The ancestral haplotypes to European N1a1 (haplotypes with 16147G) are more common in the Arabian Peninsula, northern Africa [3,20,23-26], with a limited expansion around the Iran, Israel, Turkey, Greece [13,19,27], and are relatively rare in Europe. The coalescence time for the whole N1a haplogroup based on synonymous mutations rates as well as coding region estimates was between 19,600-23,500 years (Table 2). Overall the distinct phylogeographic distribution of N1a subclusters and their coalescence times suggests that an initial diversification of N1a occurred in the Near East, followed by westwards dispersion of ancestors of particular subhaplogroup to southern Europe and northwards via Central Asian steppe zones to central Europe.

## Conclusions

Based on the current N1a haplogroup phylogeny and phylogeographic information on the farmer mtDNA associated subclades distribution, we suppose that the farmer lineages-DEB3, FLO1, and HAL2 might be derived from local communities and that they would have adopted the farming culture indigenously. Therefore, the results of the present study are somewhat difficult to reconcile with the hypothesis that the N1a lineages were brought into central Europe by the Neolithic farmers from the Near East by a major demic diffusion event. Moreover, the evidence from phylogeographic analysis of N1a lineages emphasizes that European farmer N1a lineages might have been originated from different sources- from eastern Europe (for N1a1a1), from Near East via southern Europe (for N1a1b and perhaps for N1a1a3), and from local central European source (for N1a1a2). It is thus clear that Neolithic farmers' migration into central Europe did not occur in a uniform way; indeed these results indicate that the Neolithic transition process was more complex in central Europe and possibly the farmer N1a lineages were brought in through the 'leapfrog' colonization process [5,28].

## Methods

### Samples collection

We collected about 3,625 blood samples from the South Asia region, including 64 from Nepal; 47 from Bhutan; 160 from Bangladesh and 3,354 from India. All DNA samples analyzed in the present study were derived from the blood samples collected with informed consent according to protocols approved by the Universities of North Bengal, West Bengal, India. We typed the mtDNA control region (HV-1 and HV-2) and found three individuals (one from Tamil Nadu-South India and other two from West Bengal-East India) which belonged to the N1a haplogroup. Of these three N1a individuals, two were chosen for complete mtDNA sequences analysis (sample No. 7 and No. 8 in figure 1). To get clear picture of the extent of farmer and published haplogroup N1a diversity, a further ten mtDNAs were chosen for complete sequence analysis. Those samples from Russian Federation (sample No.1- Kazakh, No. 9-Tatar, and No.10 & No.16 -Russia), No.2-Hungary, No.12-France, No.17-Italy, and No.11, 14, & 15 the United States of America have a European ancestry. Samples 2, 11, 12, 14, 15 and 17 were selected from the public participant pool through preliminary control region information that was gathered from two websites: Mitosearch http://www.mitosearch.org/ and mtDNA test results log database http://www.kerchner.com/cgi-bin/mtdna.cgi. We obtained informed consent from each participant to use their data for scientific study.

### Complete mtDNA sequencing

We obtained the complete mtDNA sequences via methods previously reported [9,29]. Sequencing was performed on 3130 and 3730 Genetic Analyzers (Applied Biosystems), and the resulting sequences were handled with the SeqScape (v. 2.5-Applied Biosystems) and DNASTAR software (DNASTAR, Inc., Madison, USA). Mutations were scored relative to the revised Cambridge Reference Sequence (rCRS) [30]. The complete mtDNA sequences of sample No. 1, 9, 10, and 16 were obtained by Genetics laboratory, Institute of Biological Problem of the North, Magadan, Russia. The remaining 8 mtDNAs complete sequences were done in the Laboratory for Conservation and Utilization of Bioresources, Yunnan University, Kunming, China. The complete mtDNA sequences reported in this paper have been deposited in GenBank under accession numbers GU123026, GU290206 - GU290216http://www.ncbi.nlm.nih.gov/Genbank/index.html.

### Phylogenetic analysis

Phylogenetic relationships among the complete mtDNA sequences were established using the reduced median network algorithm http://www.fluxus-engineering.com and the tree was checked manually to resolve homoplasies. In addition to our twelve complete sequences, seven published sequences were added to establish the N1a phylogeny (Figure 1, sample No.3, 4, 5, 18-Accession number EF153778, EF486519, EF486518, EF486517 [9]; No.13-EF657753 [31]; No. 6-EF660937 [32]; and No.19- EF184638 [33], respectively). The tree is rooted in haplogroup N, using the revised Cambridge reference sequence (rCRS) [30] as an outgroup.

### Phylogeographic and Coalescence analyses

To assess the farmer N1a type variation and phylogeographic relationship, we performed a detailed survey of modern published N1a types from West, Central and East European, the Caucasus, the Near East, and West, Central and South Asian groups. A total of 162 (excluded 4 public participants from United States of America) N1a haplotypes were observed and are presented in the table 1. By using a motif recognition and matching and near-matching strategy with our current N1a subclades phylogeny, all of the published N1a haplotypes were assigned to their respective N1a subhaplogroup. The subclade haplotype heterogeneity was evaluated with chi-square statistics (computer software available from http://www.quantpsy.org). The coalescence times were estimated with ρ statistics (mean divergence from inferred ancestral haplotype), and standard errors (σ) were calculated following the method of Saillard et al. [34]. Each subclade value of ρ ± σ was converted into time with different calibration rates as described by authors [35-39].

## Abbreviations

LBK: *Linearbandkeramik*; Sample ID related to archaeological sites-DEB: Derenburg; HAL: Halberstadt; FLO: Flomborn; UWS: Unterwiederstedt; ECS: Ecsegfalva.

## Competing interests

The authors declare that they have no competing interests.

## Authors' contributions

MGP, CLZ, BM, and MD performed laboratory analyses. MGP and YPZ conceived the project. MGP wrote the paper with help of YPZ, MD, TKC and BM (Boris Malyarchuk). All authors read and approved the manuscript.
